# A Closer Look at Schlemm’s Canal Cell Physiology: Implications for Biomimetics

**DOI:** 10.3390/jfb6030963

**Published:** 2015-09-21

**Authors:** Cula N. Dautriche, Yangzi Tian, Yubing Xie, Susan T. Sharfstein

**Affiliations:** State University of New York (SUNY) Polytechnic Institute, Colleges of Nanoscale Science and Engineering, 257 Fuller Road, Albany, NY 12203, USA; E-Mails: cula.dautriche@downstate.edu (C.N.D.); yitian@sunypoly.edu (Y.T.); yxie@sunypoly.edu (Y.X.)

**Keywords:** Schlemm’s canal, conventional outflow tract, intraocular pressure, glaucoma, biomimetics, tissue engineering, nanofabrication

## Abstract

Among ocular pathologies, glaucoma is the second leading cause of progressive vision loss, expected to affect 80 million people worldwide by 2020. A primary cause of glaucoma appears to be damage to the conventional outflow tract. Conventional outflow tissues, a composite of the trabecular meshwork and the Schlemm’s canal, regulate and maintain homeostatic responses to intraocular pressure. In glaucoma, filtration of aqueous humor into the Schlemm’s canal is hindered, leading to an increase in intraocular pressure and subsequent damage to the optic nerve, with progressive vision loss. The Schlemm’s canal encompasses a unique endothelium. Recent advances in culturing and manipulating Schlemm’s canal cells have elucidated several aspects of their physiology, including ultrastructure, cell-specific marker expression, and biomechanical properties. This review highlights these advances and discusses implications for engineering a 3D, biomimetic, *in vitro* model of the Schlemm’s canal endothelium to further advance glaucoma research, including drug testing and gene therapy screening.

## 1. Introduction

**Figure 1:**
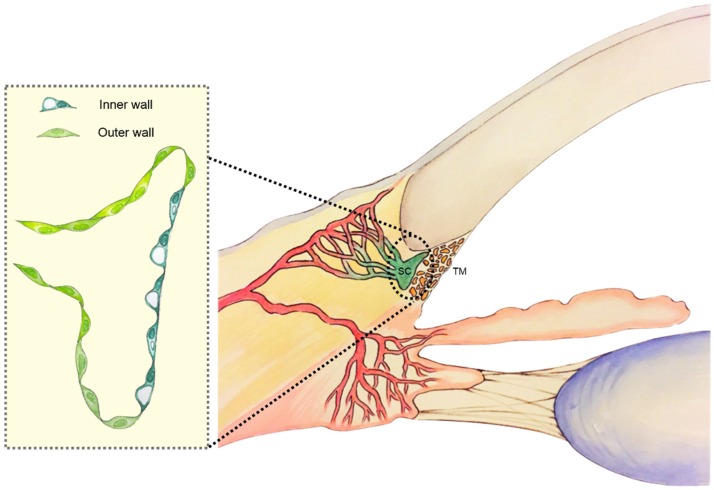
Schematic of the conventional outflow pathway. The left inset shows an expanded view of the Schlemm’s canal’s microanatomy detailing the cell morphology of the inner and outer wall.

The Schlemm’s canal (SC), named after the German anatomist, Friedrich Schlemm and first identified in 1830 [1], is a unique, ring-shaped, endothelium-lined vessel that encircles the cornea [2,3] (Figure 1). Anatomically, it is situated directly against the juxtacanalicular (JCT) region of the trabecular meshwork (TM). As a consequence, one of its primary functions is to deliver aqueous humor into the collecting channels, following filtration through the TM. Because of its close apposition to the JCT, not all SC cells are created equal. As a result, the SC is divided into the inner and outer wall, each possessing endothelial cells that differ in morphology [4], cell-specific marker expression [5,6], specialized cellular organelles, and functions (Table 1). However, these differences may be due to the differences in biomechanical environment between the inner and outer wall, rather than any underlying biological or biochemical differences between the inner and outer wall endothelia. The inner wall has been more extensively studied, as the greatest resistance to aqueous humor outflow is generated in or close to the SC endothelium that lines the TM [7,8,9,10,11]. Excessive resistance leads to elevated intraocular pressure (IOP), the leading modifiable risk factor for glaucoma. This review will focus on the development, anatomy, biology and physiology of SC inner wall endothelial cells as they are relevant to engineering the SC inner wall.

The inner wall of the SC is a unique endothelium, specialized to maintain aqueous humor homeostasis, and IOP regulation in conjunction with the TM. Unlike the TM, controversy remains regarding the SC, especially in terms of development, its contribution to outflow resistance in normal and glaucomatous states, and its role in ocular immunity. This is, in part, due to the very limited amount of SC tissue available as well as the difficulty in isolating SC cells. Moreover, 2D culture of SC cells on tissue culture plastic results in dedifferentiation of the SC cells [12], limiting its utility as a clinical model. Advances in nanotechnology, particularly materials science, have permitted cultures of SC cells in more biomimetic environments, leading to significant advances in characterizing SC cell mechano-biology and physiology, which highlights the extremely dynamic nature of the inner wall [13,14].

**Table 1:** Characteristics of Schlemm’s canal endothelial cells.

Property	Inner Wall	Outer Wall
Morphology	Cobblestone appearance [15]	Smooth and flat [5], continuous basement membrane [17]
	Discontinuous basement membrane [15,16]	
Cell-specific marker	Zipper-like VE-cadherin [18]	Desmin
		Reactivity to Factor VIII-related antigen [19]
Subcellular structure	Giant vacuoles [20], paracellular pores [21]	Weibel-Palade bodies [17]
Function	Aqueous humor filtration	Unknown
	IOP homeostasis [5,22,23]	

## 2. Embryological Origin: Of Lymph or Blood?

**Figure 2:**
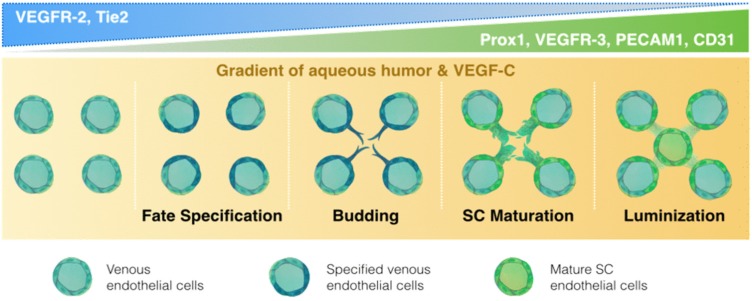
Organogenesis of the Schlemm’s canal with a focus on key differential expression pattern along with essential soluble factors.

One of the greatest controversies that surrounds the unique endothelial nature of the SC is its embryological origin. Although earlier studies suggested a vascular origin [17,24,25,26], recent publications establish the SC as a lymphatic-like vessel. In humans, prenatal SC development begins at week 17 [5] and is completed by week 24 [27], whereas in mice, the SC development is postnatal [2]. The organogenesis of the SC is a stepwise process in which SC progenitors are first specified in the transscleral veins and bud off laterally to anastomose, with subsequent lumenization and development into the mature SC (Figure 2) [28,29,30]. Park and Aspelund and their respective coworkers elucidated key molecular mechanisms and characteristics of SC progenitors for their terminal differentiation into SC cells, while contrasting it with lymphatic cell development (Figure 2). Although in mice, SC development is postnatal, unlike the embryonic development of the lymphatic system, both processes involve migration of venous endothelial cell (VEC) progenitors that undergo precise, orchestrated changes in key markers for subsequent acquisition of lymphatic identity (Table 2) [28,31].

**Table 2:** Summary of key signaling necessary for Schlemm’s canal or lymphatic development in mice.

Lineage	Development	Progenitors	Budding	Lumenization/Sac Formation	Separation from Venous Vasculature
Lymphatic	Embryonic	PROX1 [32], Sox18 [33], COUP-FII [34], VE-cadherin [35]	PDPN [36], VEFGR3 [37], CCBE1 [38], NRP2 [39], RAC1 [40], LYVE-1 [41]	NFATC1 [42], GATA2 [43,44], Calcr1 [45], Ramp2 [45], TIE1 [46]	Syk [47], SLP76 [47], Runx1 [48], PDPN [36], Meis1 [49], Clec2 [50,51], CXADR [52]
Schlemm’s Canal	Postnatal	VEGFR-2, TIE 2 [2,28]	PROX1 [2,28,55]	VEFGR-3 [2,28]	PECAM1, VEFGR-3 [2,18,28]

The SC development may be classified into four stages, SC progenitor cell-fate specification, lateral sprouting, lumenization, and separation from venous vasculature. Unlike lymphatic progenitor cell-fate specification, the key regulatory molecules and specific markers needed to mediate SC progenitor cell-fate specification have not been clearly identified. Aspelund *et al.* demonstrated that vascular endothelial growth factor (VEGF)-C is necessary to initiate migration of VECs and lateral sprouting from the transscleral veins. Park and Aspelund and their respective coworkers elegantly demonstrated that SC progenitor cells are VECs that are positive for vascular endothelial growth factor receptor (VEGFR) 2 and the tunica interna endothelial cell kinase (TIE) 2. These SC progenitor cells subsequently acquired PROX1 expression for lumenization and VEGFR-3 for subsequent maturation into SC cells [2,28] (Figure 2). Truong *et al.* were the first to demonstrate high expression of the lymphatic transcription factor, PROX1, in SC endothelium, suggesting a closer similarity between SC endothelium and lymphatic endothelium [54]. Both aqueous humor and VEGF-C are required for proper SC development. VEGF-C (VEGFc+/LacZ) heterozygous mice exhibited delayed budding of SC endothelial cells from the venous system and retarded tubular fusion [2,28]. Meanwhile, reduction of aqueous humor resulted in endothelial-mesenchymal transition and loss of the lymphatic identity [28]. Thus, the SC in mice is a unique, specialized endothelium of vascular origin that undergoes partial lymphatic reprogramming during postnatal development to acquire a transient lymphatic identity required for maintaining its proper function in aqueous humor homeostasis [2,28,55]. Similar to lymphatic cells, SC cells experience flow from a basal to apical direction. While these studies were conducted in mice, expression of PROX1 by SC endothelial cells in humans, zebrafish, and mice indicates that the lymphatic-like identity of the SC is conserved in vertebrate evolution [2], and suggests that similar developmental pathways are likely to occur in humans, albeit prenatally rather than postnatally.

Despite their lymphatic nature and expression of several (though not all) lymphatic markers, SC cells do not appear to have lymphatic origins. Kizhatil *et al.* recently detailed the organogenesis of the SC, which arises from the limbal vascular plexis (LVP) and radial vessels (RV) deep in the limbus that run in a direction perpendicular to the LVP. They coined the term canalogenesis to describe this process [53]. Canalogensis, the authors argued, is very similar to vascular development, emphasizing a more vascular origin or identity of the SC cells. However, there are important differences between angiogenesis and canalogenesis. In canalogenesis, following endothelial sprouting and tip cell formation, tip cells migrate into an intermediate zone between the LVP and RV to interact and adhere to each other, forming clusters of tip cells. The cells in these clusters divide, producing a chain of cells which acquire PROX1 expression for formation and remodeling into a tube, which is the SC. They further demonstrated that specification of the inner and outer wall of the SC is established during development with differential expression of key markers such FLT4 and PROX1.

Understanding the exact molecular footprint of SC organogenesis is at its infancy. However, these studies have radically advanced our knowledge of organogenesis of the SC. Still, important questions remain to be investigated to understand the critical contribution of the SC to aqueous humor homeostasis and glaucoma pathogenesis. For example, what determines the number of SC progenitors that will bud from the transscleral veins? What is the exact molecular footprint of SC progenitors? What triggers aqueous humor influx into the SC? What is the role of aqueous humor in the acquisition of the SC phenotype? What additional key regulators and signaling pathways are likely to participate in SC progenitor differentiation and maturation? What are the molecular events that facilitate separation from the venous system? What factors specify the cell fate of endothelial cells in the inner wall and the outer wall of the SC? Answers to these questions will facilitate establishment of platforms for manipulating SC progenitor cells to address the scarcity of SC cells available for research as well as further our understanding of human Schlemm’s canal inner wall (HSCIW) cell biology and physiology.

## 3. Schlemm’s Canal Anatomy

### 3.1. Macroarchitecture

The SC is located at the drainage or iridocorneal angle. The iridocorneal angle is lined by the TM, which overlies the SC. Together, they make up the conventional outflow tract and account for 50%–90% of aqueous humor outflow [22,54,56,57,58]. The SC is an endothelium-lined circular canal with branching of several aqueous channels. Until recently, the anatomy of the SC was characterized by histological stains, which estimated the SC cross-sectional area to be 1709 μm^2^ [58]. Recent, live, 3D, non-invasive visualization has facilitated more detailed and physiological measurement of the SC. As a result, the cross-sectional area is now estimated to vary between 4064 to 7164 μm^2^ [59,60,61,62,63], with many branched aqueous channels [64,65] (Figure 1).

### 3.2. Microarchitecture

The macroarchitecture of the SC dictates its microanatomy. The SC is lined by a continuous endothelium with tight junctions, which is divided into an outer and inner wall with regards to its relationship to the JCT (Figure 1). The SC endothelium that lies directly against the JCT is known as the inner wall and is the most celebrated and studied. The remaining endothelia comprise the outer wall. The endothelial cells of the inner wall differ from that of the outer wall in morphology, cell-specific markers and functions. In contrast to the outer wall, inner wall endothelial cells lie on a discontinuous basement membrane [15,66] and are specialized to handle flow in a basal-to-apical direction like lymphatic endothelium. The endothelial cells of the outer wall are differentiated from the inner wall endothelia by the presence of Weibel-Palade bodies [17], a positive desmin stain [67], and strong reactivity to Factor VIII-related antigen [17]. Because of their location against the JCT, the inner wall endothelial cells experience a unique biomechanical microenvironment that subjects them to a basal-apical pressure gradient. As a consequence, endothelial cells of the inner wall exhibit pores and giant vacuoles, as well as F-actin arrangements that are distinct from that of the outer wall [68]. Outer wall endothelial cells have stellate actin arrangements throughout much of the cell as compared to prominent peripheral F-actin bands observed in inner wall endothelial cells [68]. Giant vacuoles are not intracellular structures, but rather deformations of the inner wall to create a small potential space between the extracellular matrix (ECM) of the JCT and the inner wall [5]; whereas pores are inner wall structures with sizes between 0.6 and 3 μm [13] that mediate aqueous transport into the SC and may account for the SC contribution to aqueous outflow [21,69,70,71,72].

Two types of pores have been identified and characterized, I-pores (transcellular) and B-pores (paracellular) [21,73], which differ in location, sensitivity to strain and mechanisms of formation [13]. While B-pores result from local disassembly and widening of intercellular junctions, I-pores may be a result of fusion of the apical and basal cell membranes that may come into apposition as the cytoplasm thins under applied strain, with caveolae, vesicles, or “mini-pores” [13,74,75]. In addition, Braakman *et al.* recently illustrated aqueous outflow segmentation mediated by these pores, mainly B-pores [23]. Glaucomatous eyes exhibit decreased density of these pores, highlighting the vital role of the inner wall in aqueous humor homeostasis. Therefore, a goal of SC-targeted therapies might be to increase pore density and hence outflow, thus lowering IOP in glaucoma.

## 4. Characteristics of Human Schlemm’s Canal Cells

The cobblestone appearance of the HSCIW cells is attributed to the significant biomechanical load experienced as well as the segmental flow of aqueous humor [5,76]. Segmental flow relates to the non-homogenous filtration of aqueous humor in the JCT, with greater flow occurring through certain portions of the TM and less through other portions, which has been attributed to the presence or absence of pores within the HSCIW [23,77,78]. The degree of biomechanical stress directly affects the morphology of the inner wall, as its endothelial cells are described as elongated and aligned to the longitudinal axis of the SC, with some flattened and some with dome-like outpouchings (giant vacuoles) [13,57]. Because of their unique development, SC cells share morphological characteristics and cell marker expression with both lymphatic and venous endothelial cells (Table 3). In conventional 2D culture, SC cells are characterized as a homogeneous and elongated monolayer. The characteristic monolayer exhibits a net transendothelial electrical resistance of 10 Ω·cm^2^ or greater [79], an absence of myocilin induction by dexamethasone, and expression of vascular endothelial cadherin (VE-cadherin), integrin α6, and fibulin-2 [79,80]. *In vivo*, SC cells are positive for PROX1 (with much higher levels for HSCIW cells than outer wall cells), integrin α9, and CD31, but negative for the differentiated lymphatic markers LYVE-1 and podoplanin, as well as the blood vessel marker SMA [2,28,55,79,80]. The cytoskeleton of SC cells is enriched in both microfilaments and intermediate filaments, and has a prominent actin-enriched cell cortex [81]. Although traditional 2D culture systems allow for manipulation of the SC endothelial cells, SC cells in traditional culture systems usually lose essential signaling, both mechanical and biochemical, required for proper maintenance of their *in vivo* phenotypes [5,79], reducing the utility of information obtained from such systems. Therefore, 3D culture systems may promote *in vivo*-like SC morphology, marker expression and function.

**Table 3:** Comparison of Schlemm’s canal, lymphatic, and vascular endothelial properties.

Molecular/Cellular Characteristics	Schlemm’s Canal Endothelium ^a^	Lymphatic Endothelium	Vascular Endothelium
Sox18	–	+ [33]	–
VEGFR-2	+ [2,28]	–	+ [82]
VEGFR-3	+ [2,28]	+ [38]	+ [83]
PROX1	+ [2,28,53]	+ [34]	–
CCL21	+ [2,28]	–	–
Itga9	+ [2]	–	–
Collagen IV	+ [2]	–	–
PECAM1	+ [18]	–	+ [84]
VE-cadherin	+ [18]	+ [35]	+ [85]
Endomucin	+ [53]	–	–
Foxc2	+ [2,28]	–	–
LYVE-1	–	+ [41]	–
Podoplanin	–	+ [36]	–
vWF	+ [17]	–	+ [86]
Wiebel-Palade bodies	+ [17]	–	+ [86]
Endothelial monolayer	continuous [5]	continuous [29,87]	continuous
Basement membrane	discontinuous [5]	discontinuous [29,87]	continuous
Basal-to-apical Flow	+ [5,88]	+ [87,89]	–

^a^ Note that these studies did not distinguish between inner and outer wall endothelia.

We recently highlighted the importance of providing the proper 3D spatial and biochemical cues in engineering a 3D SC *in vitro* model [90]. We demonstrated that 3D culture of HSC cells on microfabricated scaffolds with well-defined physical and biochemical cues, rescued expression of key HSC markers, such as VE-cadherin and PECAM1, and mediated pore formation, crucial for the SC regulation of IOP. Whether the *in vivo* SC has been functionally or structurally replicated or even completely simulated remains to be determined with studies of physiological and structural responses to drugs and modulation of genes expression for genes such as VEGF-C.

## 5. Biomechanics

As a result of its location against the JCT, the HSCIW experiences a biomechanical microenvironment that is much closer to that of lymphatic endothelia than that of vascular endothelia. Similar to lymphatic endothelium, the HSCIW endothelia experience a basal to apical pressure gradient during aqueous outflow. Unlike lymphatic endothelium, the HSCIW endothelium is sealed by tight junctions, and thus, must support the basal-to-apical pressure drop between IOP and episcleral venous pressure, which tends to deform HSCIW cells off their supporting basement membrane, creating giant vacuoles [89,91,92,93,94]. As a result, HSCIW cells are highly contractile [95] with an estimated elastic modulus of 1–3 kPa, similar to, but somewhat larger than other endothelial cells [88,96]. In addition, as a consequence of the basal-to-apical flow, HSCIW cells also exhibit transcellular and paracellular pores to mediate aqueous humor transport [21,97,98,99]. Their unique location against the JCT subjects HSCIW endothelial cells to biomechanical signals from the JCT’s ECM [100], causing modification of gene expression to accommodate changes in substrate stiffness. HSCIW cells stiffen in response to increasing substrate stiffness, with glaucomatous HSCIW cells being more sensitive to substrate stiffness and having a larger stiffening response [88]. HSCIW cells’ ability to adapt to large deformations and respond to their microenvironment is reflected in a cytoskeletal arrangement enriched in actin microfilaments and intermediate filaments [81]. Clearly, the HSCIW’s biomechanical microenvironment plays an important role in maintaining HSCIW cells phenotype and proper function.

## 6. Perspective on Schlemm’s Canal Engineering

Although conventional 2D tissue culture is currently the primary system for evaluating and characterizing HSC cell properties, its limitations have severely impeded our understanding of trabecular outflow physiology as well as glaucoma pathology and drug screening. Currently, there is no glaucoma therapy that lowers IOP via mechanisms that target the physiology of HSCIW endothelial cells. This is, in part, due to our poor understanding of the pathology at the SC, particularly the HSCIW during glaucoma development as well as the lack of an *in vitro* system for 3D culture of these cells under flow conditions, which is necessary to capture their *in vivo* characteristics and obtain relevant clinical information. The remainder of this review will highlight the main challenges and opportunities in establishing cellular microenvironments for engineering 3D HSCIW constructs, including sources of HSCIW cells, biomaterials to mimic ECM, and soluble factors to direct and maintain functional HSCIW differentiation. 

### 6.1. Criteria for a 3D in Vitro Model of the Schlemm’s Canal Inner Wall

Until recently, HSC cell culture has been limited to traditional 2D culture or culture on microporous Transwell^®^ inserts. These studies have resulted in a tremendous amount of information on cell biology, physiology, and biomechanics. More importantly, these studies have highlighted the limitations of current systems as well as the critical attributes that a 3D *in vitro* system should recreate to correlate well with the *in vivo* characteristics and physiologic cellular response. From these studies, it is clear that any *in vitro* 3D model of the HSCIW should perform the following functions:

(1) Express key cell-specific markers, necessary for the endothelial integrity and mechanosensing;
(2) Display both paracellular and transcellular pathways vital to aqueous outflow function of the SC;
(3) Mimic the *in vivo* cellular micro architecture with respect to morphological features such as a cellular dimensions or surface area of cell-cell interactions within the cultured monolayer, the spatial distribution of subcellular organelles (vacuoles), the complexity of tight junctional strands;
(4) Allow for ease of culture using phenotypically stable cell lines to facilitate high throughput screening. Thus, a well-characterized *in vitro* 3D model of the HSCIW would provide a system in which to study and understand the physiology, biomechanics, outflow functions, physiological drug responses as well as pathological processes in glaucoma.

### 6.2. Potential Sources of Human Schlemm’s Canal Inner Wall Endothelial Cells

**Figure 3:**
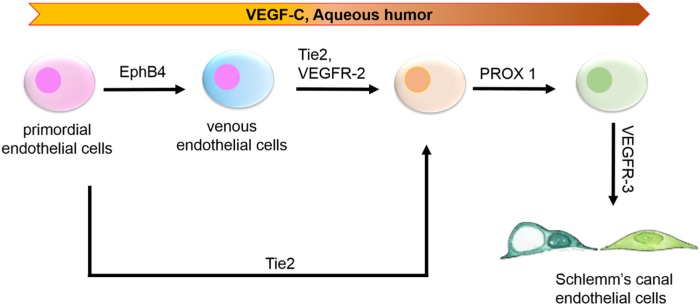
Potential strategies for stem cell differentiation into Schlemm’s canal endothelial cells.

One of the biggest challenges facing SC inner wall engineering is the scarcity of these cells [101]. Selective isolation of HSC cells from the limited amount of corneoscleral remnants remains an art. To date, very few laboratories [6,79,102,103] have developed complex protocols for successful isolation and culture of these cells and have become the primary supply sources for HSC cells, which will certainly contain both inner and outer wall HSC cells. In addition, successful isolation of these cells depends on a variety of uncontrollable factors, such as the age of the donor, duration of storage of the tissue after surgical removal before cell isolation, *etc.* These challenges have dramatically hindered the availability of SC cells for research and speak to the need to identify new ways of obtaining these cells. Stem cell differentiation [104,105,106] is an attractive avenue to explore as an alternative way of obtaining HSC cells with possible selection for HSCIW cells. Although stem cell differentiation to generate TM cells has been successfully documented [107,108,109,110], there are no reports addressing SC differentiation from stem cells. Recent publications [2,28,55] on the organogenesis of the SC have highlighted key factors and signaling molecules (e.g., PROX1 [32], VEGFR-3) that are necessary for acquisition of the SC phenotype from transscleral veins. These recent studies suggest the possibility of using primordial endothelial cells and/or venous endothelial cells [111,112,113,114] as a strategy to obtain HSCIW cells through directed differentiation (Figure 3).

### 6.3. Biomaterials for 3D Culture of Human Schlemm’s Canal Inner Wall Cells

In addition to the scarcity of HSC cells, challenges in conventional 2D culture of HSC cells have impeded our understanding of the functional contribution of the SC to outflow physiology and glaucoma pathology. HSC cells in conventional 2D culture are distinct from their *in vivo* counterpart as they lose expression of key *in vivo* cell-specific markers [18,79,80]. This dedifferentiation speaks to the need to engineer an *in vitro* model of the SC that can mimic the *in vivo* microenvironment, eventually capturing the 3D *in vivo* characteristics of these cells. Given that the SC inner wall layer is only a few microns thick [96,115], a top-down approach to engineering the inner wall may be the most feasible strategy. In the traditional top-down approach, exogenous biocompatible and mechanically competent scaffolds are fabricated for 3D culture of the cells, which are then allowed to populate the scaffold, deposit and remodel their ECM.

Scaffolds are fundamental to tissue engineering. Their functions include providing mechanical support, supporting ECM production and cell colonization, and waste-nutrient exchange [116,117,118]. As a consequence of its endothelial origin, scaffold materials and fabrication techniques being used in vascular tissue engineering can provide insight into engineering the SC [119,120,121,122] although different geometries will be required due to the unique nature of the SC canal, in particular, the polarization between in the inner and outer wall. Successful biomaterials for scaffolding for SC engineering might incorporate synthetic polymers, for their mechanical strength and well controlled porosity [123], and natural polymers, for their biochemical cues. In our previous work, we adapted the negative photoresist, SU-8, to provide the necessary topographical and mechanical cues while using the hydrogel Extracel™ to promote cell attachment and maintenance of SC cells differentiated functions [90].

Micro and nanofabrication techniques, such as lithography and electrospinning, are versatile fabrication techniques widely used in production of fibrous and porous scaffolds for vascular tissue engineering. Given the porous nature of the conventional outflow tract, scaffold considerations for SC engineering should be tailored to mimicking the pore structures (e.g., pore size, porosity), and in particular, extracellular, biochemical, and biomechanical microenvironments of the region directly against the SC, the JCT. Pore sizes and ECM fiber diameters in the JCT range from 2 to 15 μm [16]. Thus, scaffolds with various permutations of these properties might facilitate 3D culture of SC cells. In addition, biochemical cues play a paramount role in maintaining cell phenotype and function. The JCT ECM components, by virtue of their location against the HSCIW, might be providing key biochemical signaling for HSCIW cell growth and function. Thus, natural polymers of ECM components [8,124] like those found in the JCT, such as hyaluronic acid and collagen IV can be used to provide biochemical cues for HSCIW cell growth and function. Therefore, microfabricated scaffolds of synthetic polymers can be surface-coated or chemically modified with ECM components found at the JCT-SC border to provide key biochemical cues for successful HSCIW engineering. ECM components, such as hyaluronic acid [125,126,127] and collagens [128,129,130], have been used to support endothelial cell proliferation and function, indicating their potential to modify microfabricated scaffolds for HSCIW engineering. Additionally, the stratification of these ECM components at the JCT-SC interface highlights their potential as scaffolding materials or scaffold supplements for 3D HSCIW cell culture.

### 6.4. Soluble Factors for Directed Schlemm’s Canal Cell Differentiation

Cellular differentiation is a result of coordinated dynamic expression of hundreds of genes and proteins in precise response to external signaling cues [106,131], which include soluble factors and spatio-physical cues from the ECM. Soluble factors mediate cellular differentiation by binding to cell surface receptors, thus activating downstream signaling [132]. Lineage specification of soluble factors, of the same stem cell type, differs depending on whether the stem cells were cultured in 3D or 2D configurations. For example, when induced to differentiate in restrictive ECM environments, adhesive, flattened human mesenchymal stem cells (hMSCs) in 2D preferentially adopt an osteogenic phenotype, whereas round hMSCs in 3D cultures preferentially undergo adipogenesis [133,134,135]. Thus, soluble factors and spatio-temporal cues in 3D culture are vital in providing the microenvironment necessary for differentiation, and may favor one lineage over another.

Park and Aspelund and their respective coworkers have elegantly elucidated key molecular footprints of venous endothelial cell differentiation to SC cells. Together, their data highlights the essential role of soluble factors such as VEGF-C, VEGF-D and aqueous humor for venous endothelial cells to acquire SC cell identity and for proper development of the SC. In addition, these data suggest the possibility of using these factors to mediate differentiation of induced pluripotent stem cells into SC cells [136]. Given the intimate relationship of the HSCIW to the JCT and that TM development precedes SC development, it is equally likely that soluble factors from the JCT cells may be vital to acquiring and maintaining the SC/HSCIW phenotype. Several groups have documented that cytokine (TNF-α, IL1-α, IL-β, and IL-8) release by TM cells mediates SC cell function in regulating aqueous humor outflow [100,137,138], highlighting the possible role of TM cells and their soluble factors in SC differentiation. Nitric oxide (NO) has been extensively studied for its role in modulating SC cell behavior to regulate aqueous outflow [3,103,139]. Because of the important role that NO plays in facilitating SC cell functions and endothelial junctional integrity, this warrants exploring the role of NO in SC development [140]. Furthermore, given the vascular origin of the SC and its lymphatic-like development and characteristics, it is important to consider soluble factors involved in vascular endothelial and lymphatic cell differentiation. Because of the unique biomechanical environment of the SC, direct addition of soluble factors to a 3D culture of the HSCIW cells may not be sufficient. Hence, controlled and sustained delivery of these soluble factors may be crucial for successful differentiation. Thus, other approaches such as nanoparticle-based delivery or conjugation to nanofibers that facilitate timed and spatial release should be consider for the delivery of soluble factors to induce SC cell differentiation and organogenesis [141,142,143,144,145].

### 6.5. Dynamic 3D Culture

The *in vivo* forces generated by aqueous humor flow play an important role in the SC organogenesis [2,28]. They are vital in maintaining the morphology and physiology of the HSCIW cells. Several groups have attempted to replicate the dynamic microenvironment of HSCIW cells *in vitro* through culture on microporous Transwell^®^ membranes [100,146,147,148]. Together these studies have highlighted possible mechanisms for aqueous humor transport across the HSCIW, namely via formation of giant vacuoles and paracellular pores. They have further demonstrated the important role of the pressure gradient in modulating the barrier function of the HSCIW through regulation of junctional proteins. While these studies were able to capture the *in vivo* cell polarization and provided tremendous information regarding the biomechanics of the HSCIW, this system is not ideal to study aqueous outflow, or to perform continuous perfusion, medium exchange or gradient studies, particularly in response to key signaling factors like VEGF-C. These limitations are due to the nature of Transwell^®^ membranes, which are track-etched and possess irregular pore structures or have low porosity (*i.e.*, 4%–20%), indicating poor topographical approximation [149] and limiting their performance for assessing physiologic parameters [150]. In addition, culture of endothelial cells on Transwell^®^ membranes results in a less stringent endothelial barrier with the occurrence of irregular patterns of cell adhesion or “edge effect” [151], hampering the integrity of the endothelial monolayer. Thus, culture of HSCIW cells on Transwell^®^ membranes to assess physiologic parameters uniquely associated with these cells may limit the clinical relevance obtained from such system*s.* Therefore, culture methods which incorporate a dynamic flow element might be instrumental not only for HSCIW cell differentiation, but for proper simulation and maintenance of the HSCIW phenotype *in vitro*. Dynamic flow system such as direct perfusion bioreactors and microfluidic devices that enable sophisticated control of the spatial, temporal profile of gradients as well as flow velocities are more suitable to culture of SC cells.

The *in vitro* system we previously described overcame some of the limitations of commercially available Transwell^®^ membranes. Using photolithography techniques, we fabricated highly porous SU-8 membranes with pre-defined porosity, well-controlled, uniform pore size, shape, and beam width [90,152,153], demonstrating that well-defined SU-8 scaffolds support a more *in vivo*-like SC morphology, characterized by re-expression of key *in vivo* endothelial markers, PECAM1 and VE-cadherin, pore formation and outflow function. This system is a major step forward in the culture of SC cells, but it does not simulate the *in vivo* structure, in terms of fluidic or mechanical stress [90]. More studies are needed exploring 3D cultured cells in systems that can better simulate their *in vivo* dynamic microenvironment [154]. For instance, several studies have documented the importance of mimicking the dynamic microenvironment in lymphatic endothelial cell culture, demonstrating the critical role of interstitial flow in modulating lymphatic endothelial cell proliferation, migration and function [155,156]. In the case of cardiac cell differentiation, culture methods that incorporate a dynamic flow element improve cardiogenesis, beating percentage in size-controlled hESC-derived embryoid bodies (EBs), and cardiac gene expression at mRNA and protein levels in mESC-derived EBs [157,158,159,160] when compared to traditional static culture methods [161]. Therefore, in a similar fashion, replicating the dynamic microenvironment via continuous perfusion through SC cells might result in differences in cellular morphology, junctional complex expression and formation, and even outflow regulation. In addition, commercial Transwell^®^ membranes are not ideal for co-culture, as they are too thick to allow for necessary cell-cell communications and their low porosity for appropriate rate of nutrients and paracrine signal exchange. Given that the HSCIW is in close apposition to the JCT, the most optimal culture system for both the SC cells and trabecular meshwork cells, is a co-culture on a membrane thin enough to allow for direct cell-cell communication, paracrine signals and a membrane strong enough to withstand appropriate flow velocities while under continuous perfusion. Thus, the fluid dynamics of the SC must be captured *in vitro*, for proper simulation of the *in vivo* tissue to obtain more clinically relevant responses, especially for drug studies.

## 7. Conclusions

The SC is a unique vascular endothelium with lymphatic-like characteristics that functions to mediate IOP outflow homeostasis together with the TM. The exact contribution of the SC is yet to be delineated. This is partly due to the lack of an *in vitro* model that can facilitate 3D culture of HSCIW cells, recapturing their *in vivo* phenotype and thus, obtaining more physiologically relevant information. Research efforts targeting the engineering of the conventional outflow tract and its components are in their infancy. Applying nanotechnology for engineering the conventional outflow tract has great potential to mimic the nanoscale structure and outflow function of HSCIW cells. If successful, 3D culture of HSCIW cells will provide a valuable *in vitro* model that may revolutionize current thinking on the contribution of the SC to conventional outflow tract physiology and pathology and will hopefully translate into new drug modalities for glaucoma, targeting the SC.

## Abbreviations

Calcr1	Calcitonin Receptor 1
CCBE1	Collagen and calcium-binding EGF domain-containing protein 1
CCL21	Chemokine (C-C motif) ligand 21
Clec2	C-type lectin-like receptor 2
COUP-FTII	Chicken ovalbumin upstream promoter-transcription factor 2
CXADR	Coxsackie virus and adenovirus receptor
Foxc2	Forkhead box protein C2
GATA2	GATA binding protein 2
Itga9	Integrin alpha-9
LYVE-1	Lymphatic vessel endothelial hyaluronan receptor
Meis1	Meis homeobox 1
Nfatc1	Nuclear factor of activated T-cells, cytoplasmic 1
NRP2	Neuropilin 2
PDPN	Podoplanin
PECAM1	Platelet endothelial cell adhesion molecule
PROX1	Prospero homeobox protein 1
RAC1	Ras-related C3 botulinum toxin substrate 1
Ramp2	Receptor activity modifying protein 2
Runx1	Runt-related transcription factor 1
SLP76	Lymphocyte cytosolic protein 2
Syk	Spleen tyrosine kinase
TIE1	Tunica interna endothelial cell kinase 1
TIE2	Tunica interna endothelial cell kinase 2
VE-cadherin	Vascular endothelial cadherin
VEFGR-2	Vascular endothelial growth factor 2
VEFGR-3	Vascular endothelial growth factor 3
vWF	Von Willebrand factor
